# Supplementary material for: Mechanisms governing the pioneering and redistribution capabilities of the non-classical pioneer PU.1
Source: Nat Commun. 2020 Jan 21;11:402. doi: 10.1038/s41467-019-13960-2 (PMC6972792; doi:10.1038/s41467-019-13960-2)
Supplement: Supplementary file 7 — Source data [file 41467_2019_13960_MOESM7_ESM.zip › Source_Data/FigureS2/FigureS2B_MotifScanOutput/KmeansCluster7/homerResults/motif10.similar.html]

motif10

## Information for motif10

C
T
G
A
C
G
T
A
C
G
A
T
C
G
T
A
A
C
T
G
C
T
A
G
A
T
C
G
C
G
T
A
C
G
T
A
A
T
G
C
  
Reverse Opposite:  

A
T
C
G
A
C
G
T
A
C
G
T
A
T
G
C
A
G
T
C
A
G
T
C
C
G
A
T
C
G
T
A
C
G
A
T
A
G
C
T
  

|  |  |
| --- | --- |
| p-value: | 1e-109 |
| log p-value: | -2.511e+02 |
| Information Content per bp: | 1.837 |
| Number of Target Sequences with motif | 339.0 |
| Percentage of Target Sequences with motif | 10.56% |
| Number of Background Sequences with motif | 939.3 |
| Percentage of Background Sequences with motif | 2.15% |
| Average Position of motif in Targets | 98.7 +/- 51.1bp |
| Average Position of motif in Background | 101.0 +/- 77.4bp |
| Strand Bias (log2 ratio + to - strand density) | 0.3 |
| Multiplicity (# of sites on avg that occur together) | 1.02 |
| Motif File: | file (matrix) reverse opposite |

### Similar de novo motifs found

|  |  |  |  |  |  |  |  |
| --- | --- | --- | --- | --- | --- | --- | --- |
| Rank | Match Score | Redundant Motif | P-value | log P-value | % of Targets | % of Background | Motif file |
| 1 | 0.758 | G T C A T A G C C G A T C G A T T G A C C G A T T A G C C G A T G C T A G C A T C A G T | 1e-34 | -80.420005 | 2.49% | 0.34% | motif file (matrix) |
| 2 | 0.860 | G C T A T G C A C T G A C G A T C G T A A T C G C T G A A T C G C G T A C G T A A G T C G T C A | 1e-15 | -35.598692 | 0.87% | 0.09% | motif file (matrix) |
